# First Molecular Characterization of *Trueperella pyogenes* Isolated from a Rabbit Periodontal Abscess

**DOI:** 10.3390/vetsci12060573

**Published:** 2025-06-11

**Authors:** Magdalena Kizerwetter-Świda, Ewelina Kwiecień, Ilona Stefańska, Dorota Chrobak-Chmiel, Magdalena Rzewuska, Wojciech Bielecki

**Affiliations:** 1 Department of Preclinical Sciences, Institute of Veterinary Medicine, Warsaw University of Life Sciences, Ciszewskiego 8, 02-786 Warsaw, Poland; ewelina_kwiecien1@sggw.edu.pl (E.K.); magdalena_rzewuska@sggw.edu.pl (M.R.); 2 Department of Pathology and Veterinary Diagnostics, Institute of Veterinary Medicine, Warsaw University of Life Sciences, Nowoursynowska 166, 02-787 Warsaw, Poland

**Keywords:** *Trueperella pyogenes*, rabbit, periodontal abscess, pyolysin, virulence factors

## Abstract

Periodontal abscesses are a frequent health problem found in pet rabbits that could lead to severe complications, including life-threatening ones. *Trueperella pyogenes* is an opportunistic pathogen isolated from pyogenic infections in different animal species, mostly from farm animals such as ruminants and pigs. This study presents the first case of isolation and molecular characterization of *T. pyogenes* from a periodontal abscess in a pet rabbit. A narrative review concerning the bacteriological aetiology of odontogenic abscesses in rabbits is also presented. The identification of *T. pyogenes* isolate was obtained based on the phenotypic properties and confirmed by molecular biology methods, i.e., the 16S rRNA gene sequencing and the presence of species-specific pyolysin gene. Furthermore, the pathogenic potential of the isolate was confirmed by the detection of six out of eight studied virulence genes. This report, along with the literature data, confirms that *T. pyogenes* may be one of the etiological agents of periodontal abscesses in rabbits. However, inadequate laboratory techniques may result in an underestimation of the occurrence of this bacterial species in rabbits. This study showed the need for standardization of the laboratory methods used for extended microbial culture for the clinical samples obtained from periodontal abscesses in rabbits. These procedures should also include the isolation of fastidious pathogens like *T. pyogenes*.

## Simple Summary

Periodontal abscesses represent a common and significant health issue in rabbits. Here, we describe the first case of isolation and molecular characterisation of *Trueperella pyogenes* from a pet rabbit. The identification was obtained by phenotypic properties and molecular biology techniques, including detection of the species-specific pyolysin gene and 16S rRNA gene sequencing. We detected six out of eight studied virulence genes, underscoring the pathogenic potential of this isolate. Our findings and the existing literature suggest that fastidious bacteria like *T. pyogenes* are significant etiological agents in rabbit periodontal abscesses. This study highlights the need for the standardisation of the laboratory methods used for extended microbial culture for the clinical samples of rabbit origin, particularly from periodontal abscesses.

## 1. Introduction

Periodontal abscesses, a common health issue in pet rabbits, are caused by different factors, such as diet, trauma, infections, metabolic bone diseases, or neoplasma. However, they are usually related to dental problems [1,2,3]. Despite the frequent occurrence of such cases, the literature data regarding bacteriological aetiology are sparse. Available publications indicate that both aerobic and anaerobic bacteria may be isolated from rabbit abscesses in rabbits. The most frequently obtained bacteria are *Pasteurella multocida*, *Staphylococcus aureus*, *Streptococcus* spp. and *Pseudomonas* spp., while *Bacteroides* spp. and *Fusobacterium* spp. predominate among anaerobic bacteria [1,4,5,6]. *Trueperella pyogenes* is a facultatively anaerobic, opportunistic pathogen isolated from purulent infections in different animal species, occasionally from humans. These infections are reported in farm and free-living animals, with most documented cases concerning cattle and pigs [7,8,9,10]. Recently, there have been reports regarding the isolation of *T. pyogenes* from unusual animal hosts such as grey slender lorises (*Loris lydekkerianus nordicus*), okapi (*Okapia johnstoni*) and royal python (*Python regius*) [11,12].

The paper aimed to present an unusual aetiology of periodontal abscess in a pet rabbit. To our knowledge, this is the first description of a *T. pyogenes* isolate obtained from a rabbit, including its phenotypic characterization, molecular species identification and the detection of virulence genes.

## 2. Materials and Methods

### 2.1. Isolation and Phenotypic Identification of Trueperella pyogenes

The clinical material for bacteriological examination was obtained from a 6-year-old male intact French lop rabbit (*Oryctolagus cuniculus*). Visible head deformations in the maxillary and mandibular area were the reason for the visit to the veterinary clinic. The rabbit was kept in a household with no other animals, in a standard cage, with ad libitum access to commercial feed and hay. Clinical examination revealed the presence of two masses of the right cheek and left mandibular area. Oral examination showed instability of the left lower premolar (P1). After its extraction, a fistula leading to a subcutaneous abscess in the left mandibular area was detected. The abscess was swabbed and the material was submitted for routine bacteriological examination. Treatment included local procedures (curettage and drainage) and antibiotic therapy according to the results of antibiotic susceptibility testing for isolated bacteria.

A swab taken from the abscess was inoculated on Columbia Agar with 5% sheep blood (CA) (Graso Biotech, Starogard Gdański, Poland) and McConkey Agar (Graso Biotech, Starogard Gdański, Poland). Incubation was carried out in aerobic conditions and 5% CO_2_ atmosphere at 37 °C for four days. Additionally, to isolate anaerobic bacteria Schaedler Broth (Graso Biotech, Starogard Gdański, Poland) medium with paraffin was inoculated and incubated for 48 h. The isolated microorganism was subjected to a routine bacteriological identification, i.e., Gram staining method, catalase test, CAMP test with the reference strain *Staphylococcus aureus* ATCC 25923 and API Coryne test (bioMérieux, Franc).

### 2.2. Antimicrobial Susceptibility Testing

The isolate obtained from the rabbit was subjected to antimicrobial susceptibility testing (AST) using the disk diffusion method [13]. The suspension density of bacterial cells in sterile saline was adjusted to a 0.5 McFarland standard and spread evenly on Mueller Hinton agar (Graso Biotech, Starogard Gdański, Poland) supplemented with 5% sheep blood. The disks with the following antimicrobial agents were applied: penicillin (P), cefpodoxime (CPD), clindamycin (CC), tetracycline (CC), enrofloxacin (ENR), chloramphenicol (C) and sulfamethoxazole with trimethoprim (SXT). After incubating at 37 °C with 5% CO_2_ for 24 h, the diameters of growth inhibition were measured with a calliper. The recommendations for the interpretation of AST are not available for *T. pyogenes*. Thus, breakpoints for *Streptococcus* spp. were used, and *Streptococcus pneumoniae* ATCC 49619 was used as a quality control [14].

### 2.3. 16S rRNA Gene Sequence Analysis

A genomic DNA for molecular biology tests was obtained using the boiling method. A few colonies from a pure culture of the isolate cultured on CA were suspended in 200 µL of nuclease-free water. Then, the suspension was boiled for 10 min, cooled on ice and centrifuged (5 min, 8000× *g*). The supernatant was used as a DNA template for PCR. The 16S rRNA gene sequencing confirmed the phenotypic identification of the isolate. A fragment of the 16S rRNA (843 bp) gene was amplified using universal primers UNF (5′-GAGTTTGATCCTGGCTCAG-3′) and UNR (5′-GGACTACCAGGGTATCTAAT-3′) [15].

In order to perform phylogenetic analysis, the 16S rRNA gene sequence obtained from the isolate described in this study was compared with 18 sequences of the 16S rRNA gene of *T. pyogenes* isolates isolated from different hosts (pig, calf, goat, sheep, deer, *Bison bonasus*, *Naemorhedus goral*) in various countries (Poland, China, Japan), along with three type strains deposited in the GeneBank, i.e.,: *T. pyogenes* ATCC 19411, *Trueperella bernardiae* ATCC BAA-441 and *Trueperella bialowiezensis* DSM 17162. The analysis also included the sequence of *Arcanobacterium haemolyticum* DSM 20595 as additional references. All sequences were aligned using The MEGA 12.0 package [16]. Multiple sequence alignment of the partial 16S rRNA gene sequences from 19 *T. pyogenes* strains showed similarity ranging from 100% to 99.08%. The phylogenetic tree was reconstructed using the neighbour-joining method [17], with bootstrap analysis based on 1000 replications [18]. The evolutionary distances were computed using the maximum composite likelihood method [19].

### 2.4. Detection of Virulence Genes

The presence of the following virulence determinants was investigated: pyolysin, neuraminidase H, neuraminidase P, four fimbrial subunits (A, C, E and G) and collagen-binding protein. The primers and amplification conditions were described by Silva et al. (2008) [7]. *T. pyogenes* ATCC 49698 was used as a reference strain. All amplification products were visualised by electrophoresis in 1.5% (*w*/*v*) agarose gel.

## 3. Results

### 3.1. Isolation and Phenotypic Identification of Trueperella pyogenes

**Figure 1:**
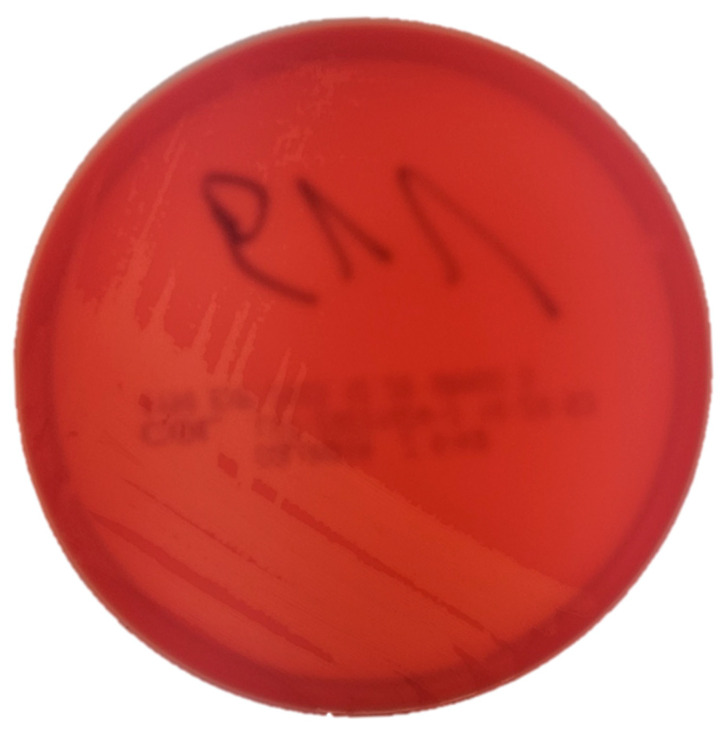
Colony morphology of *Trueperella pyogenes* isolated from the rabbit on Columbia Agar with 5% sheep blood, 48 h of incubation in CO_2_ atmosphere at 35 °C.

After 48 h of incubation on a CA in 5% CO_2_ atmospheric conditions, tiny β-hemolytic colonies with a diameter of less than 1 mm were detected (Figure 1). After 72 h of incubation, the colonies were approximately 1 mm in diameter, white, round and showing β-hemolysis. Similar, white, tiny β-hemolytic colonies, about 1 mm in diameter on CA medium incubated in aerobic conditions, appeared after four days of incubation. After two days of incubation, slight turbidity of the Schaedler’s medium under the paraffin was observed. Subsequently, a subculture was made from Schaedler’s medium to CA medium, on which, after 48 h of incubation in 5% CO_2_ atmosphere at 37 °C, tiny β-hemolytic colonies were found, with the morphology described above (diameter of less than 1 mm). No microbial growth was observed on McConkey Agar. No other bacteria were cultured.

The morphology of bacterial cells was assessed using the Gram staining method revealed Gram-positive irregular rods. The test for catalase production was negative. In the CAMP test with the reference strain *Staphylococcus aureus* ATCC 25923 a positive result was observed, visible as the enhancement of β-hemolysis. The isolated microorganism was identified using the API Coryne test as *T. pyogenes*.

### 3.2. Antimicrobial Susceptibility Testing

The examined *T. pyogenes* isolate showed a high level of susceptibility to antimicrobial agents, i.e., penicillin, cefpodoxime, clindamycin, tetracycline, enrofloxacin and chloramphenicol. Resistance was shown only to sulfamethoxazole with trimethoprim (Table 1).

### 3.3. 16S rRNA Gene Sequence Analysis

**Figure 2:**
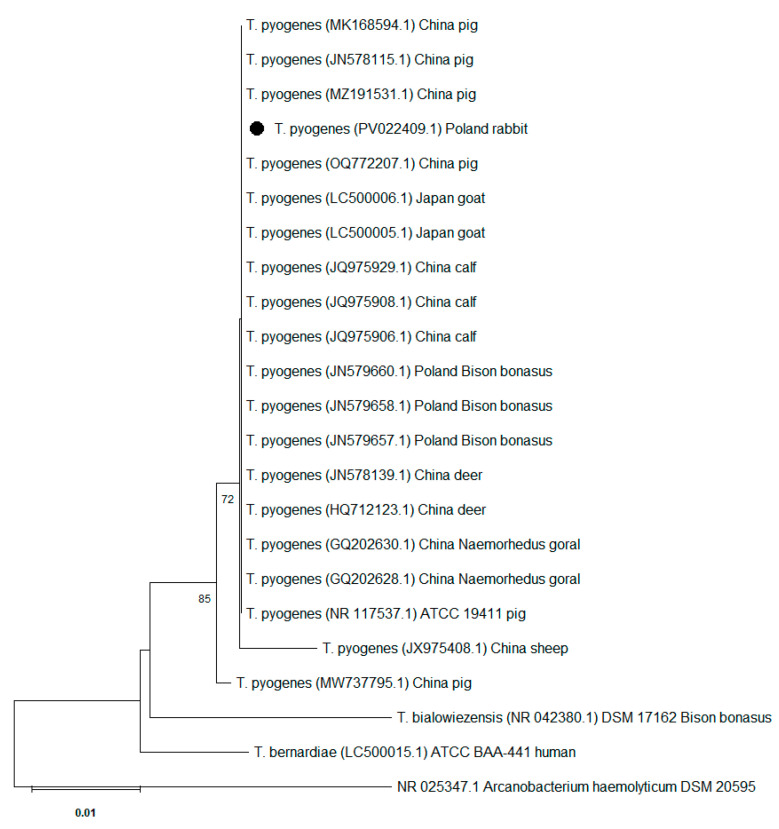
Phylogenetic tree based on partial sequences of 16S rRNA gene of the *Trueperella pyogenes* isolate obtained from rabbit (marked with a dot), *T. pyogenes* obtained from different hosts in various countries deposited in the GenBank (NCBI) and reference strains of *T. pyogenes* ATCC 19411, *Trueperella bernardiae* ATCC BAA-441 and *Trueperella bialowiezensis* DSM 17162. The tree was constructed using the neighbour-joining method of 16S rRNA gene sequences. Numbers at branch nodes represent the percentage of replicate trees in which the associated taxa clustered together in bootstrap tests (1000 replicates). *Arcanobacterium haemolyticum* DSM 20595 was used as an outgroup. Bootstrap values below 70 are not shown. The scale bar represents 0.01-nucleotide substitutes per position.

The obtained 16S rRNA gene sequence has been deposited in the NCBI GenBank (accession number PV022409). The GenBank BLAST (v2.16.0) analysis of the sequence showed 100.00% identity with sequences of several *T. pyogenes* strains deposited in the NCBI database, including *T. pyogenes* strain ATCC 19411 (GeneBank accession number NR117537.1), finally confirming the identification of the tested isolate as *T. pyogenes*. Based on partial 16S rRNA gene sequences, the phylogenetic analysis indicated a close genetic relationship between the different *T. pyogenes* isolates included in the analysis. *T. pyogenes* isolate obtained from a rabbit was clustered with other isolates included in the analysis and with the reference strain, *T. pyogenes* ATCC 19411 (Figure 2).

### 3.4. Detection of Virulence Genes

**Figure 3:**
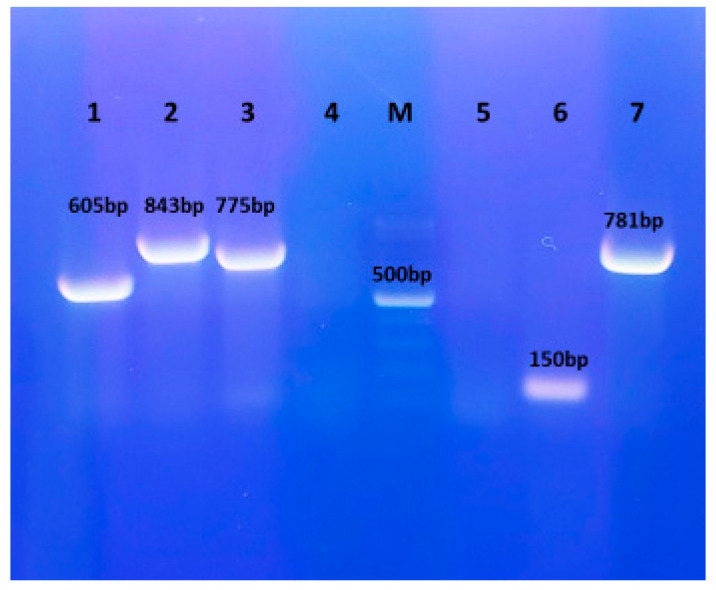
Agarose gel electrophoresis of the virulence genes in *Trueperella pyogenes* isolate obtained from a rabbit. Line 1: *fimA* (605 bp); Line 2: *fimC* (843 bp); Line 3: *fimE* (775 bp); Line 4: *fimG* (negative); M—molecular weight marker; Line 5: *cbpA* (negative); Line 6: *nanP* (150 bp); Line 7: *nanH* (781 bp). Positive results were obtained in lines 1–3 and 6 and 7. Negative results were obtained in lines 4 and 5.

Six of eight tested virulence determinants were detected in the *T. pyogenes* isolate of rabbit origin. The presence of the pyolysin gene and three fimbrial subunit genes (*fimA*, *fimC* and *fimE*) was confirmed. Additionally, both neuraminidase genes (*nanH* and *nanP*) were found. The *fimG* gene and collagen-binding protein gene were not detected (Figure 3).

## 4. Discussion

This study presented an abscess in a rabbit of an unusual aetiology. To the best of our knowledge, there are no reports describing the isolation of *Trueperella pyogenes* from rabbits, along with molecular identification and characterization of the isolated strain. Five reports on the isolation of *T. pyogenes* from clinical materials obtained from rabbits are available, but the identification was based only on phenotypic properties. One publication described a case of severe infection with the formation of multiple abscesses around the stifle joint and the sternum. The authors could not determine the source of this unusual infection, even though this rabbit was kept in a laboratory animal husbandry, where contact with the external environment is limited. The bacteriological examination of material collected from abscesses showed the presence of *T. pyogenes*, although the identification was based only on phenotypic methods [20]. Two further publications concern the retrospective analysis of microbiological culture results. One of them is a retrospective analysis of the results of bacteriological examinations of clinical materials collected from rabbits over the years 2010–2021 in Spain, which mentions *T. pyogenes* as one of the rarely isolated species from abscesses and dental diseases, in 32 (1.1%) out of 3596 cases. The authors performed a study on results obtained from a private diagnostic laboratory, and the detailed culture methods were not disclosed in this publication [21]. Moreover, two reports were published demonstrating the isolation of *T. pyogenes* from suppurative disorders in rabbits. One concerning lung lesion in an animal with septicemia, in the other, bacteria were isolated from the uterine content in rabbits with pyometra [22,23]. The latest report from 2024 provided an analysis of the medical record databases regarding rabbits with dental diseases (2013–2023), in which 51 cases were included. Bacterial culture conditions were not described, only one *T. pyogenes* isolate was found [24].

In our study *T. pyogenes* was the only bacterial species isolated from a rabbit abscess, indicating its involvement in the infection. The result of bacterial culture in the CO_2_ atmosphere was visible after 48 h in the form of tiny colonies. The interpretation of culture results on Schaedler’s broth under paraffin was possible only after subculture onto a CA agar, i.e., after four days of incubation. Bacterial growth in aerobic conditions was delayed and detectable after four days of incubation. These results confirm that the use of 24 h or 48 h of aerobic incubation is not effective for the culture of *T. pyogenes*, as well as other fastidious microorganisms.

The obtained isolate was characterized by the presence of genes encoding virulence factors, among which the *plo* gene is regarded as the most important in pathogenicity and also species-specific [8]. Thus, the detection of the *plo* gene confirms the phenotypic identification. Other virulence genes detected in the isolate include fimbrial subunits genes, *fimA*, *fimC* and *fimE*, and two neuraminidase genes, *nanH* and *nanP*. Two virulence factor genes were not detected: the *fimG* and the *cbpA*. *T. pyogenes* isolates obtained from different animals, and from various types of infection, expressed different virulence factor genes, except the *plo* gene, which was always detected. Different profiles of virulence genes were observed in *T. pyogenes*, with the *fimG*, *cbpA* and neuraminidase genes being detected the least often [7,9]. The detection of six out of eight virulence factors in the isolate described in this study indicates its high pathogenic potential.

The phylogenetic analysis showed high homology of the 16S rRNA gene sequence in the tested isolate obtained from the rabbit and in isolates belonging to this species originating from different hosts and isolated in different countries as well as to the reference strain *T. pyogenes* ATCC 19411. This is consistent with the results indicating high similarity of the 16S rRNA sequences and the lack of specificity of *T. pyogenes* for different host species [25,26].

Although odontogenic abscesses in rabbits are a common health problem leading to morbidity and in case of complications even to mortality, the literature data regarding this issue are relatively scarce. Available research results on the bacterial aetiology of periodontal abscesses in rabbits indicate that they are often mixed infections involving both aerobic and anaerobic bacteria. Some discrepancies in the literature result from differences in methods used to collect clinical materials and microbiological culture conditions [5,6,27]. Different culture bacterial methods are used in individual studies, which makes it impossible to compare the results. If aerobic culture conditions are used, *Pasteurella multocida, Staphylococcus* spp., *Streptococcus* spp. and *Pseudomonas* spp. are most frequently found. The anaerobic species isolated from these lesions were predominantly *Bacteroides* spp. and *Fusobacterium* spp. [1,4,5]. Moreover, the bacteriological culture results interpretation may be hampered by isolating a few bacterial species, including faecal microorganisms. Coprophagia, a physiological behaviour in rabbits, leads to the presence of faecal bacteria in the oral cavity, most of which cause opportunistic infections [27]. However, if several bacterial species are isolated from an abscess, distinguishing whether they solely represent contamination of the clinical material becomes challenging. Furthermore, antibiotic therapy against faecal bacteria may lead to gastrointestinal tract dysbiosis and severe diarrhoea. Another essential element during the collection of the clinical materials from the oral cavity is to avoid contamination by gingival biota or dental plaque bacteria. Incorrect collection of clinical specimens for microbiological culture may lead to inconclusive results [4]. An additional factor that may influence the effectiveness of treatment procedures is the thick consistency of pus. Lysosomal enzymes involved in the digestion of dead cells and purulent material liquefaction are not produced by rabbits. The aspiration or drainage of pus from the abscess may be difficult, as the penetration of antimicrobials [4].

In the study of abscesses from 12 rabbits described by Tyrrell et al. (2002), aerobic and anaerobic culture conditions were used, and two samples from each rabbit were examined: a biopsy specimen with the abscess margin and bone (if available) [4]. The second specimen was pus from the centre of the abscess. In this study, *Fusobacterium nucleatum* (6/12), *Micromonas micra* (5/12) and *Streptococcus intermedius* (6/12) were most frequently isolated, and in individual cases various species of aerobic and anaerobic bacteria were obtained. Interestingly, *P. multocida*, *S. aureus* and *Bacteroides fragilis* were not detected. Another publication described the results of a retrospective analysis of microbiological examinations of 48 rabbits with dental abscesses [5]. In most cases (73.3%), aerobic and anaerobic bacteria were isolated, and in 50.8% of cases more than three bacterial species were obtained. A total of 185 isolates were obtained, including 52 aerobic bacteria and 133 anaerobic species. The unusual domination of anaerobic species may result from the accidental isolation of contaminating faecal bacteria. The most common bacteria isolated in this study included *Pseudomonas aeruginosa* (14/52), *Pasteurella* spp. (10/52), *Streptococcus* spp. (8/52) *Staphylococcus* spp. (7/52), *Fusobacterium* spp. (36/133), *Peptostreptococcus* spp. (27/133) and *Bacteroides* spp. (27/133). A comparison of the results presented in the works of Tyrrell et al. (2002) and Gardhouse et al. (2017) is complicated by the different methods used for collecting clinical samples [4,5]. Tyrell et al. (2002) employed percutaneous excision of abscesses to avoid contamination with gingival bacteria [4]. In the second study, swabs were collected from the oral cavity and the inside of the abscesses [4].

A study describing the outcome of wound-packing treatment of dental abscesses in 13 rabbits was published by Taylor et al. (2010) [1]. Additionally, the bacteriological examination of purulent materials was performed using culture conditions for aerobic and anaerobic bacteria. Mixed infections caused by aerobic and anaerobic bacteria were generally found, but the authors did not provide detailed culture conditions or incubation time. However, there was no bacterial growth in 4 out of 14 (28.6%) abscesses, despite their presence in direct smears. It can be assumed that appropriate methods of culture for the fastidious microorganisms, including *T. pyogenes,* were not used *T. pyogenes*.

Periodontal infections often are caused by endogenous oral bacteria that penetrate the surrounding tissues [27]. Thus, it is advisable to determine the composition of the natural oral microbiota in healthy animals, but only one publication concerning rabbits is available in the literature. In research conducted by Flenghi et al. (2023), the bacterial microbiota of 33 healthy pet rabbits was evaluated using culture methods for aerobic and anaerobic bacteria [27]. However, an essential drawback of this study is the short incubation time of only 24 h, which is insufficient for some microorganisms, especially anaerobic ones and *T. pyogenes*. This may explain the results obtained, where aerobic bacteria, such as *Streptococcus* spp, were most often isolated. (19.8%), *Rothia* spp. (17.9%), *Enterobacter* spp. (7%) and *Staphylococcus* spp. (6.6%). The genera of bacteria isolated from healthy individuals were mainly in concordance with those obtained from abscesses by other authors, except for *Rothia* spp. and *Enterobacter* spp., found only in healthy rabbits [27]. Another study concerning the aetiology of bacterial infection in rabbits (n = 170), where the majority were dental diseases (n = 60). However, conditions for the isolation of fastidious and anaerobic bacteria were not included [6].

There is a significant limitation of our study. Unfortunately, due to the owner’s decision, the follow-up visit did not take place. Thus, it was impossible to re-examine the rabbit and assess the effectiveness of the applied treatment procedures.

## 5. Conclusions

This study demonstrated that *T. pyogenes* may be isolated from periodontal abscesses in rabbits. However, further studies are required to confirm its prevalence in rabbits. Moreover, the review of the sparse and inconsistent literature data indicates the need for standardization of microbiological culture methods regarding the clinical samples from periodontal abscesses in rabbits. The methodology should include culture conditions for aerobic and anaerobic bacteria, as well as the prolonged time of incubation for *T. pyogenes* and some other fastidious bacterial species.

## Figures and Tables

**Table 1:** The results of antimicrobial resistance testing with the disk diffusion method of *Trueperella pyogenes* isolate described in this study.

Antimicrobial	Disk Content	Diameter of Growth Inhibition Zone (mm)	Interpretation
Penicillin	10 U	25	S
Cefpodoxime	10 µg	26	S
Clindamycin	2 µg	22	S
Tetracycline	30 µg	28	S
Enrofloxacin	5 µg	25	S
Chloramphenicol	30 µg	24	S
Sulfamethoxazole with trimethoprim	23.75/1.25 µg	10	R

S—susceptible, R—resistant.

## Data Availability

The data generated and analyzed during the current study are available in the NCBI GenBank repository, under the accession number: PV022409.

## References

[1] Taylor W.M., Beaufrère H., Mans C., Smith D.A. (2010). Long-term outcome of treatment of dental abscesses with a wound-packing technique in pet rabbits: 13 cases (1998–2007). J. Am. Vet. Med. Assoc..

[2] Levy I., Mans C. (2024). Diagnosis and outcome of odontogenic abscesses in client-owned rabbits (*Oryctolagus cuniculus*): 72 cases (2011–2022). J. Am. Vet. Med. Assoc..

[3] Rätsep E., Ludwig L., Dobromylskyj M. (2024). Orofacial masses in domestic rabbits: A retrospective review of 120 cases from 2 institutions, 2000–2023. J. Vet. Diagn. Investig..

[4] Tyrrell K.L., Citron D.M., Jenkins J.R., Goldstein E.J. (2002). Periodontal bacteria in rabbit mandibular and maxillary abscesses. J. Clin. Microbiol..

[5] Gardhouse S., Sanchez-Migallon Guzman D., Paul-Murphy J., Byrne B.A., Hawkins M.G. (2017). Bacterial isolates and antimicrobial susceptibilities from odontogenic abscesses in rabbits: 48 cases. Vet. Rec..

[6] Crăciun S., Novac C.Ş., Fiţ N.I., Bouari C.M., Bel L.V., Nadăş G.C. (2025). Bacterial Diversity in Pet Rabbits: Implications for Public Health, Zoonotic Risks, and Antimicrobial Resistance. Microorganisms.

[7] Silva E., Gaivão M., Leitão S., Jost B.H., Carneiro C., Vilela C.L., Lopes da Costa L., Mateus L. (2008). Genomic characterization of *Arcanobacterium pyogenes* isolates recovered from the uterus of dairy cows with normal puerperium or clinical metritis. Vet. Microbiol..

[8] Rzewuska M., Kwiecień E., Chrobak-Chmiel D., Kizerwetter-Świda M., Stefańska I., Gieryńska M. (2019). Pathogenicity and Virulence of *Trueperella pyogenes*: A Review. Int. J. Mol. Sci..

[9] Kwiecień E., Stefańska I., Kizerwetter-Świda M., Chrobak-Chmiel D., Didkowska A., Bielecki W., Olech W., Krzysztof Anusz K., Rzewuska M. (2022). Prevalence and Genetic Diversity of *Trueperella pyogenes* Isolated from Infections in European Bison (*Bison bonasus*). Animals.

[10] Kwiecień E., Stefańska I., Kizerwetter-Świda M., Chrobak-Chmiel D., Czopowicz M., Moroz-Fik A., Mickiewicz M., Biernacka K., Bagnicka E., Kaba J. (2024). Genetic diversity and virulence properties of caprine *Trueperella pyogenes* isolates. BMC Vet. Res..

[11] Nagib S., Glaeser S.P., Eisenberg T., Sammra O., Lämmler C., Kämpfer P., Schauerte N., Geiger C., Kaim U., Prenger-Berninghoff E. (2017). Fatal infection in three Grey Slender Lorises (*Loris lydekkerianus nordicus*) caused by clonally related *Trueperella pyogenes*. BMC Vet. Res..

[12] Ahmed M.F.E., Alssahen M., Lämmler C., Eisenberg T., Plötz M., Abdulmawjood A. (2020). Studies on *Trueperella pyogenes* isolated from an okapi (*Okapia johnstoni*) and a royal python (*Python regius*). BMC Vet. Res..

[13] CLSI (2017). Methods for Antimicrobial Susceptibility Testing of Infrequently Isolated or Fastidious Bacteria Isolated from Animals.

[14] CLSI (2024). Performance Standards for Antimicrobial Disk and Dilution Susceptibility Tests for Bacteria Isolated from Animals.

[15] Alexeeva I., Elliott E.J., Rollins S., Gasparich G.E., Lazar J., Rohwer R.G. (2006). Absence of *Spiroplasma* or other bacterial 16s rRNA genes in brain tissue of hamsters with scrapie. J. Clin. Microbiol..

[16] Kumar S., Stecher G., Suleski M., Sanderford M., Sharm S., Tamura K. (2024). MEGA12: Molecular Evolutionary Genetic Analysis version 12 for adaptive and green computing. Mol. Biol. Evol..

[17] Saitou N., Nei M. (1987). The neighbor-joining method: A new method for reconstructing phylogenetic trees. Mol. Biol. Evol..

[18] Felsenstein J. (1985). Confidence Limits in Phylogenies: An Approach Using the Bootstrap. Evolution.

[19] Tamura K., Nei M., Kumar S. (2004). Prospects for inferring very large phylogenies by using the neighbor-joining method. Proc. Natl. Acad. Sci. USA.

[20] Shahbazfar A.A., Kolahian S., Mohammadpour H., Helan J. (2013). Multi abscessation with multinodular abscesses in a New Zealand white rabbit (*Oryctolagus cuniculus*) following *Arcanobacterium pyogenes* infection. Revue. Méd. Vét..

[21] Fernández M., Garcias B., Duran I., Molina-López R.A., Darwich L. (2023). Current Situation of Bacterial Infections and Antimicrobial Resistance Profiles in Pet Rabbits in Spain. Vet. Sci..

[22] Hijazin M., Ulbegi-Mohyla H., Alber J., Lämmler C., Hassan A.A., Abdulmawjood A., Prenger-Berninghoff E., Weiss R., Zschöck M. (2011). Molecular identification and further characterization of *Arcanobacterium pyogenes* isolated from bovine mastitis and from various other origins. J. Dairy Sci..

[23] Planas J., Pintado E., Verdés J., Lourdes Abarca M., Martorell J. (2020). Rabbit with polyuria and polydipsia. 2020. J. Exot. Pet. Med..

[24] Minich D.J., Marrow J.C., Sadar M.J., Borsdorf M.C. (2024). High incidence of complications following intraoral extractions and treatment of periapical infections in the management of domestic rabbit (*Oryctolagus cuniculus*) dental disease (51 cases). J. Am. Vet. Med. Assoc..

[25] Rogovskyy A.S., Lawhon S., Kuczmanski K., Gillis D.C., Wu J., Hurley H., Rogovska Y.V., Konganti K., Yang C.Y., Duncan K. (2018). Phenotypic and genotypic characteristics of *Trueperella pyogenes* isolated from ruminants. J. Vet. Diagn. Investig..

[26] Magossi G., Gzyl K.E., Holman D.B., Nagaraja T.G., Amachawadi R., Amat S. (2025). Genomic and metabolic characterization of *Trueperella pyogenes* isolated from domestic and wild animals. Appl. Environ. Microbiol..

[27] Flenghi L., Mazouffre M., Le Loc’h A., Le Loc’h G., Bulliot C. (2023). Normal bacterial flora of the oral cavity in healthy pet rabbits (*Oryctolagus cuniculus*). Vet. Med. Sci..

